# Social, Environmental, and Health Vulnerability to Climate Change: The Case of the Municipalities of Minas Gerais, Brazil

**DOI:** 10.1155/2017/2821343

**Published:** 2017-03-29

**Authors:** Ana Flávia Quintão, Isabela Brito, Frederico Oliveira, Ana Paula Madureira, Ulisses Confalonieri

**Affiliations:** ^1^Escola de Saúde Pública do Estado de Minas Gerais, Belo Horizonte, MG, Brazil; ^2^Centro de Pesquisas René Rachou, Fundação Oswaldo Cruz, Belo Horizonte, MG, Brazil; ^3^Instituto Militar de Engenharia, Rio de Janeiro, RJ, Brazil; ^4^Universidade Federal de São João del Rey, São João del Rey, MG, Brazil

## Abstract

Vulnerability to climate change is a complex and dynamic phenomenon involving both social and physical/environmental aspects. It is presented as a method for the quantification of the vulnerability of all municipalities of Minas Gerais, a state in southeastern Brazil. It is based on the aggregation of different kinds of environmental, climatic, social, institutional, and epidemiological variables, to form a composite index. This was named “Index of Human Vulnerability” and was calculated using a software (SisVuClima®) specifically developed for this purpose. Social, environmental, and health data were combined with the climatic scenarios RCP 4.5 and 8.5, downscaled from ETA-HadGEM2-ES for each municipality. The Index of Human Vulnerability associated with the RCP 8.5 has shown a higher vulnerability for municipalities in the southern and eastern parts of the state of Minas Gerais.

## 1. Introduction

Vulnerability to climate change has been defined by the Intergovernmental Panel on Climate Change (IPCC) as “the propensity or predisposition to be adversely affected” [1]. In considering the perspective of scenarios of climatic anomalies vulnerability may be regarded as a possibility of “future damage” [2]. There is a consensus that vulnerability is a complex and dynamic phenomenon and that several characteristics of a given social-ecological system contribute to make people and territories more or less vulnerable [3]. These characteristics can be grouped as being related to the exposure, the sensitivity, or the adaptive capacity of the system being exposed [4].

Recent approaches to the comparative assessment of vulnerability to climate change have included the quantification of characteristics such as income, education, employment, gender, health status, mortality, physical aspects of the environment, institutional capacity, access to clean water, basic sanitation, and climatic scenarios [5–14].

**Figure 1:**
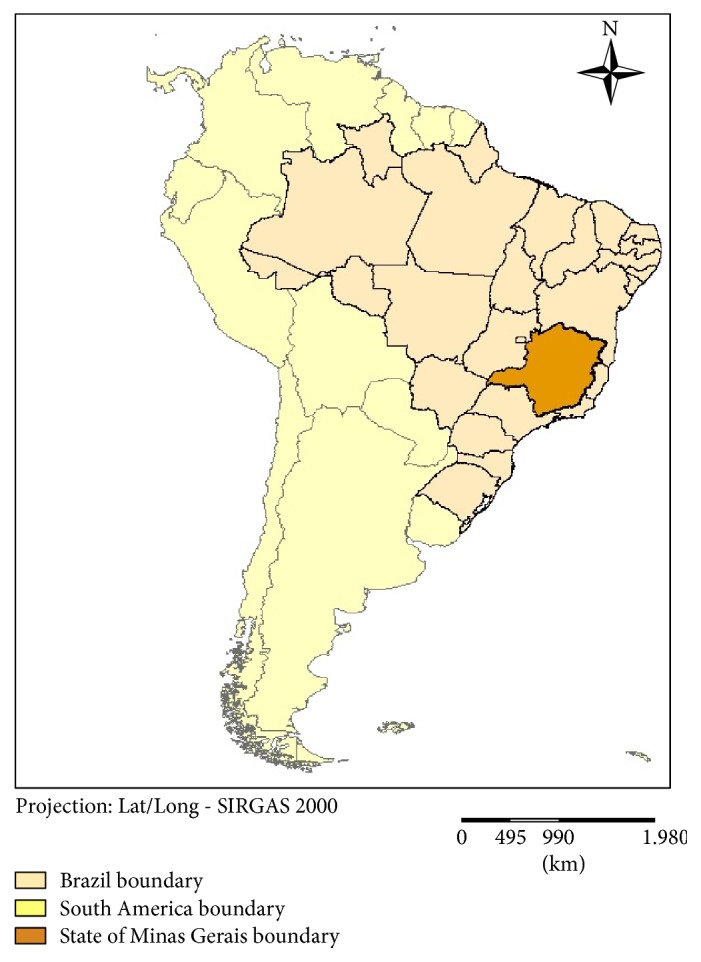
State of Minas Gerais.

In this paper we have developed and applied a system of aggregate indicators based on different sets of social, demographic, environmental, health, and climatic data from governmental sources to compare each of the 853 municipalities of the state of Minas Gerais, in southeastern Brazil (Figure 1). These sets of data were processed using software developed specifically for the purpose of obtaining and comparing vulnerability indices. The municipal indices were combined to downscale climatic scenarios for each municipality in order to obtain a standardized Human Vulnerability Index to climate change for each unit of analysis.

## 2. Methods

### 2.1. Study Area

Minas Gerais is a state in southeastern Brazil with a territory of 586,522 square kilometers and is divided into 853 municipalities which are grouped in 17 “development territories,” based on social, economic, cultural, and geographical features. This large territory includes different biomes, ranging from the semiarid dryland in north to the Atlantic Forest in the east and south (Figure 1).

Several climate-sensitive infectious diseases such dengue fever, Zika virus, leishmaniasis, and leptospirosis are endemic in Minas Gerais. The profiles of the municipalities range from small territories (3,57 sq. km) to large areas (10.727,47 sq. km), with populations ranging from 815 to 2.7 million inhabitants [15].

### 2.2. Data Analysis

The present study shows an Index of Human Vulnerability at the municipal level, under two climatic scenarios: Representative Concentration Pathways (RCP) 4.5 and 8.5. The RCP scenarios, used by the IPCC, are related to the radiative forcing over the earth's surface, as measured in Watts/m^2^ (4.5 and 8.5 W/m^2^). The RCP 4.5 assumes a less severe scenario of warming, when compared to the RCP 8.5.

The data and variables used were selected based on previous research developed by the group [9–11] as well as on the specific literature on the quantification of vulnerability and also on the availability of the data.

The Brazilian Institute for Space Research (INPE) through its Center for Weather Forecast and Climatic Studies (CPTEC) has been periodically downscaling global models for South America, from the HadGEM2-ES Model [16]. The last published version includes a 20 km grid of horizontal resolution, which was used as basis for the current study.

The climatic variables used from the model output were average daily temperatures (TP2M) in Celsius degrees and the total daily precipitation (mm). The climatic anomalies considered were the absolute difference between the future climate (2011–2040) and the 1961–1990 baseline. The spatial analysis of the climatic variables was made through geostatistical modeling using the tool “geostatistical analyst” from the two types of software ArcGis Desktop 10.2.2 and Surfer version 11. The process included the development of an experimental semivariogram for the description of the spatial dependency of the variables followed by an interpolation with ordinary kriging in order to estimate the values of the climatic variables for nonsampled localities. The last step in the process was a cross validation to assess the uncertainty associated with the parameters obtained.

The downscaled values for all municipalities of Minas Gerais have shown a general pattern of changes in average temperature and precipitation. The INPE's regional model ETA is nested in the global model HadGEM2-ES. Anomalies were calculated for two RCP: 4.5 and 8.5.

The indicators used to characterize the exposure, sensitivity, and adaptive capacity were based on governmental data sources which are available to the public and are updated periodically.

### 2.3. Software for Data Analysis

Data were analyzed using the software SisVuClima, developed by a team from Fiocruz, specifically for the calculation of vulnerability indices related to climatic change.

SisVuClima is a system developed to both calculate the indices and produce thematic maps using the cartographic base of the municipalities. The system allows the periodic updating of indices through the inclusion of new data. The registration of data was made both automatically, from existing data bank, and manually. The statistical calculation uses the “R” language from software freely available in the web. SisVuClima is compatible with both Windows and Linux operational systems [17].

### 2.4. The Conceptual Model

**Figure 2:**
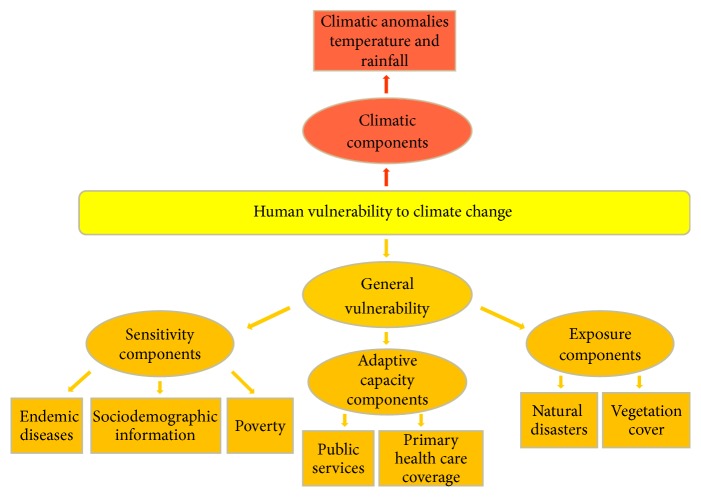
Conceptual model, source: elaborated by the author.

The approach used to produce the metric for each municipality was the development of a composite indicator called “Index of Human Vulnerability” (IHV). This is divided into three main groups of indicators, addressing the aspects related to “exposure,” “sensibility,” and “adaptive capacity” (Figure 2).

Indicators in the Exposure Index included the conservation of natural ecosystems, considered as a proxy for environmental services, and historical data showing the occurrence of weather-related disasters in each municipality. The indicators used for the sensibility component were measures of poverty, literacy, age groups, sanitation, infant mortality, and the incidence of climate-sensitive endemic infectious diseases. For the characterization of the Adaptive Capacity a combination of the FIRJAN Index and the structure of primary health care for each municipality was used (Table 1).

The FIRJAN Index of Municipal Development is produced periodically by the Federation of Industries of the State of Rio de Janeiro to assess the capacity of all municipalities of Brazil to provide basic services to its population. It includes components related to income/employment, education, and access to primary health care [18].

Indicators for each variable (or grouped variables) were ranked in classes according to their values using the* K*-means method. The indices were then standardized to vary from zero (less vulnerable) to one (more vulnerable) using the following general formula:

(1) $$\begin{array}{c} Ip=\frac{Iobs-Imin}{Imax-Imin}, \end{array}$$

Where *Ip* is standardized index, *I*obs is calculated index (for each municipality), *I*max is maximum value of the index among all municipalities, and *I*min is minimum value of the index among all municipalities.

## 3. Results

**Figure 3:**
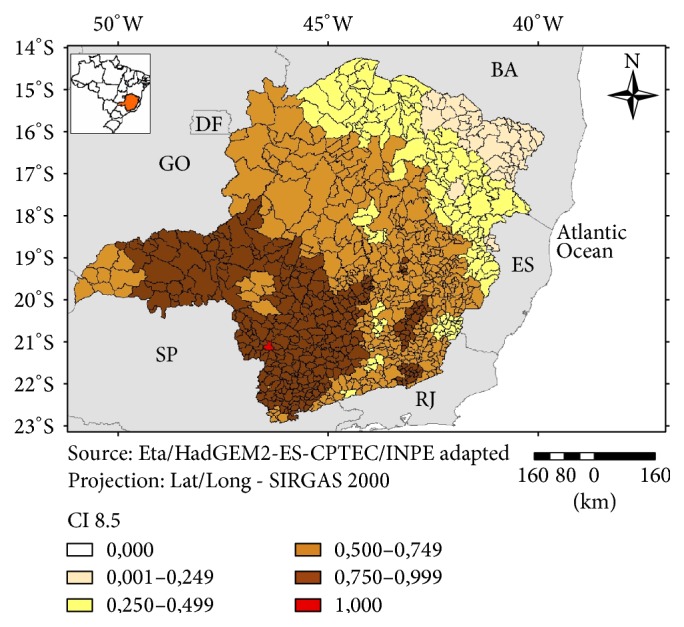
Climate Index 8.5 (CI 8.5), Minas Gerais, 2016.

**Figure 4:**
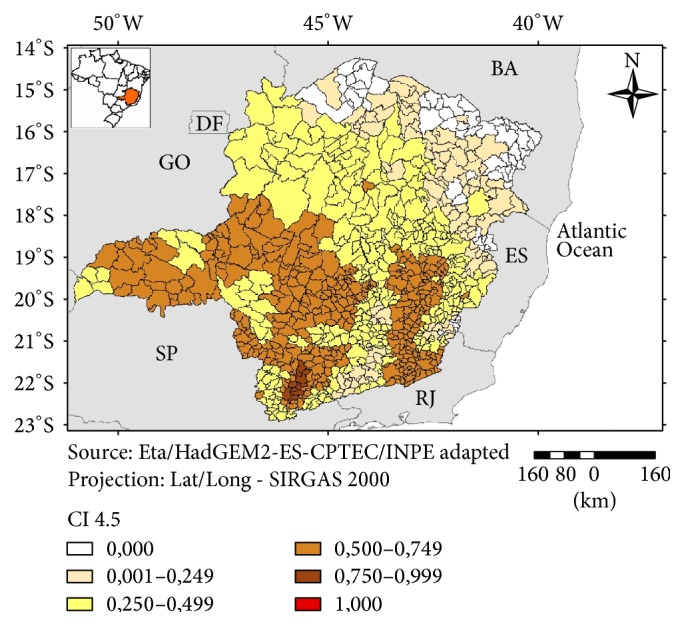
Climate Index 4.5 (CI 4.5), Minas Gerais, 2016.

A clear pattern of anomalies (temperature and precipitation) was found for RCP 8.5, with a gradual decrease in projected values starting in the southwestern part of Minas Gerais (Figure 3). For RCP 4.5 a less clear pattern of anomalies was observed (Figure 4).

**Figure 5:**
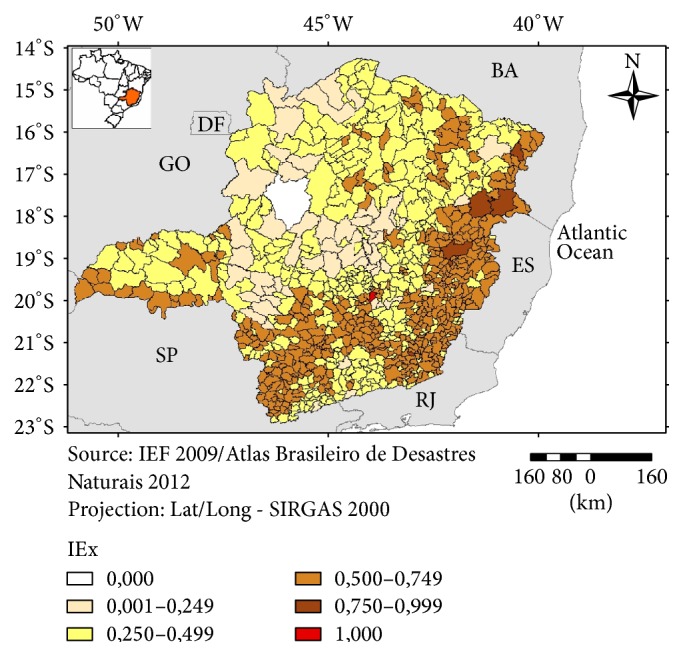
Index of Exposure (IEx), Minas Gerais, 2016.

**Figure 6:**
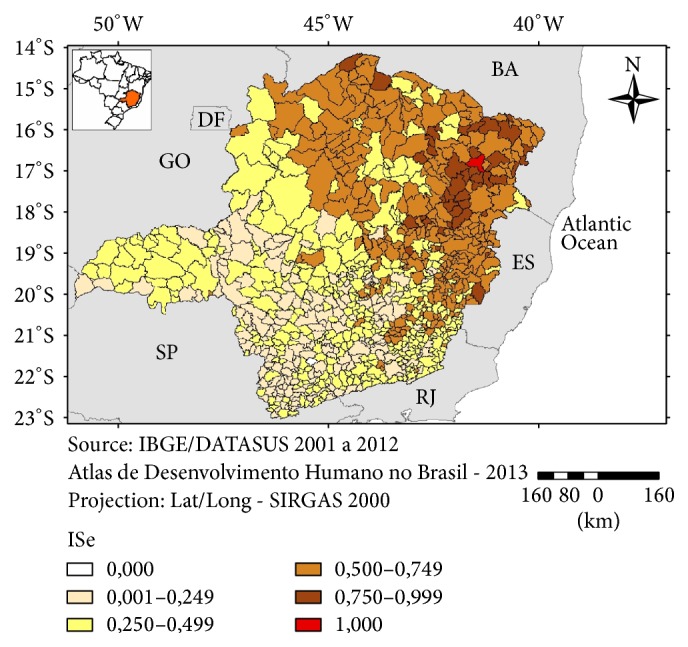
Index of Sensitivity (ISe), Minas Gerais, 2016.

**Figure 7:**
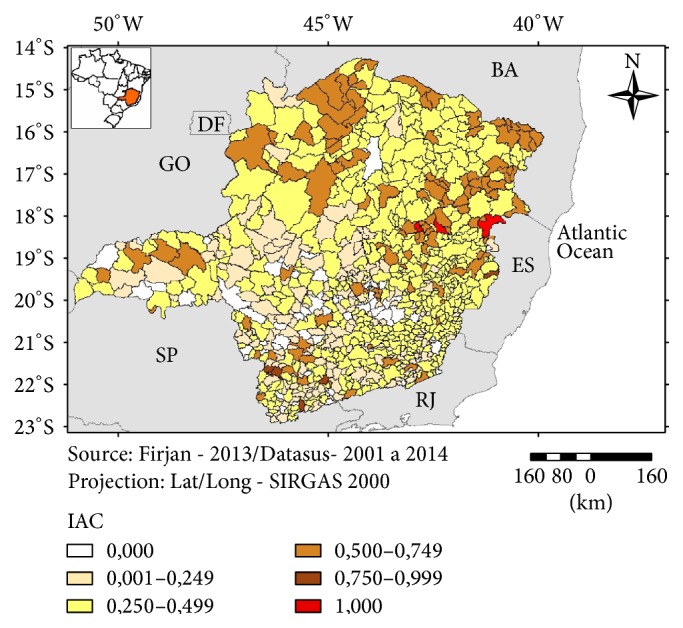
Index of Adaptive Capacity (IAC), Minas Gerais, 2016.

The exposure component of the vulnerability was more intense in the eastern and southern parts of Minas Gerais (Figure 5) whereas the sensitivity component was worse for the municipalities from the northern and northeastern parts of the state (Figure 6). The index of Adaptation Capacity did not show a clear pattern of distribution with most of the municipalities ranked as more vulnerable being scattered in the northern, western, and parts of the eastern region (Figure 7).

**Figure 8:**
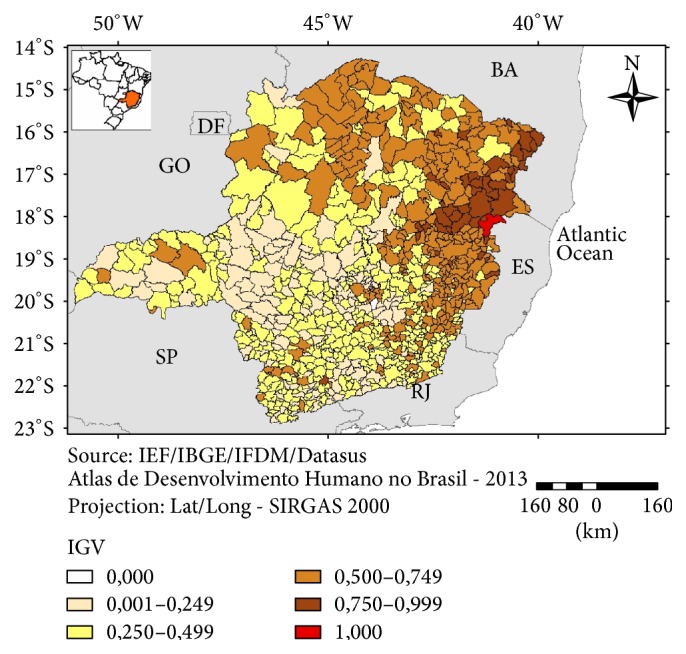
Index of General Vulnerability (IGV), Minas Gerais, 2016.

The Index of General Vulnerability (IGV), which included all variables and indicators from the three components of vulnerability (except the Climate Index), was higher in a cluster of municipalities in the northeastern part of the state and, to a lesser extent, in the northern and part of the eastern region (Figure 8). The association of this IGV with the two indicators for climate change, the Climate Index (CI) 4.5 and CI 8.5, has produced two different pictures for each scenario.

**Figure 9:**
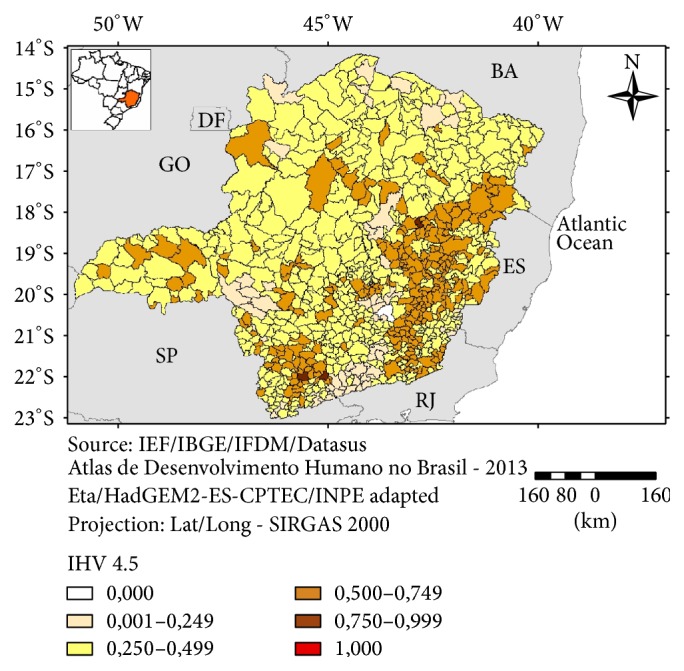
Index of Human Vulnerability (IHV 4.5), Minas Gerais, 2016.

In regard to the IHV 4.5 (Figure 9) a large group of more vulnerable municipalities was found in the eastern part of the state. There are also clusters of highly vulnerable municipalities in the northeastern and southeastern parts of Minas Gerais. These are regions with HDI ranging from medium to high [19] and, as observed in the present study, their municipalities ranked as less vulnerable in the classification of the “General Vulnerability” Index (excluding the climatic scenarios). However, the scenarios for climate anomalies have shown a more intense change for these regions both for the 4.5 and the 8.5 scenarios (Figures 3 and 4). These regions are also known historically for a high incidence of natural disasters (storms; floods) due to their mountainous topography and a reduced natural vegetation cover [20]. The sensitivity component was more important for the northeastern municipalities and the climatic anomalies indicated by the scenarios have also influenced the overall vulnerability for this region.

**Figure 10:**
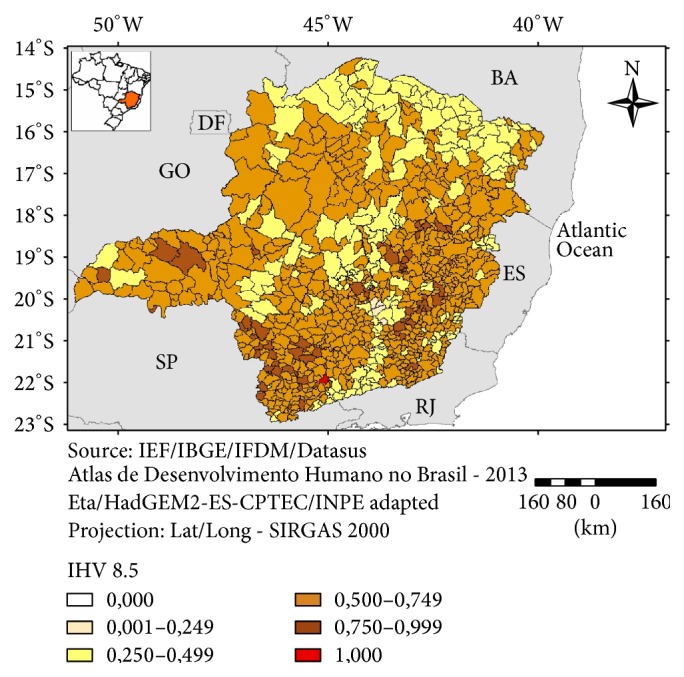
Index of Human Vulnerability (IHV 8.5), Minas Gerais, 2016.

In regard to the IHV 8.5 the distribution of the more vulnerable municipalities is more homogeneous and a clear trend was not observed, except for a small group of territories with higher vulnerabilities in the southwestern and eastern parts of the state (Figure 10). This pattern was also observed under the 4.5 scenario. A smaller number of municipalities with higher Vulnerability Index values was also observed in the western and central part of Minas Gerais, under the 8.5 scenario. These regions are considered as more developed and were compared with the other parts of the state.

## 4. Discussion

Although “vulnerability” has been commonly equated with “poverty” it has a wider meaning. In the case of the impacts of climate change vulnerability means also exposure to stresses and difficulties in managing these. That is, it depends on effective adaptive responses to risk [21, 22].

Some authors consider separately a “biophysical” from “social” component of vulnerability. The former is related to the frequency and severity of given type of hazard and the consequences of its impacts. Social vulnerability includes all intrinsic properties of a given system regardless of the pattern of exposures. Vulnerability is, therefore, the result of the interaction of the hazards of a given place with the social profile of the communities in that same place [23–25].

In the current assessment we have integrated indicators of both physical and social components of vulnerability. The physical component was represented by the indicator of vegetation cover, due to its protective effect on the environment, and indicators related to climate-associated disasters and the climatic scenarios. The retrospective incidence of climate-sensitive diseases is also part of the “physical” component and they were included since a high burden of disease increases the sensitivity of the population to the impacts of climatic variation and change. All other indicators used were selected to represent the social profile of the population at the municipalities.

In the aggregation of indicators to form the Index of Adaptive Capacity we have considered basically the capacity of the local governments to provide public services to the population, which are important for the adaptation process. This has included indicators of health services performance whereas the Index of Sensitivity has included epidemiological indicators reflecting the health status of the population. These were associated with standard measures of poverty such as income, the structure of households, literacy, and sanitation. The sensitivity component also included the proportion of young children and elderly in the population since these are generally considered as more vulnerable in the discussions on the impacts of climatic hazards [7]. All these indicators are routinely produced by the Brazilian government after the collection of data for the demographic census and other processes of population counts.

The group of indicators selected for the metric on vulnerability is publicly available and since the software SisVuClima will have open access these will allow for the technical staff of the state government to update the vulnerability indices as new data become available. The major reason for developing the tool for the updating of the calculation of the Vulnerability Index (the software SisVuClima) is the assumption that vulnerability is a dynamic entity, dependent on physical and social processes which are continuously changing [26]. The components of this Index of vulnerability can be replaced in the future as other types of more representative data, or even indicators, become available. This would be possible due to the relative easiness for operating structural changes in the SisVuClima software.

The depiction, for a given municipality, of the indicators that have contributed mostly to a higher vulnerability (e.g., through “radar” type of graphs) will allow the local administration to prioritize, based on sound information, investments for specific sectors such as sanitation and education of health infrastructure. On the other hand, by having a map of the vulnerability of all municipalities of the state (Figures 9 and 10) the state government will have a comprehensive view of the spatial distribution of the vulnerability factors in the territory under administration that will subsidize the decision making process related to public policies for social and environmental protection.

## 5. Conclusions

This study has shown high vulnerability of the human population from the northeastern and southeastern part of the Brazilian state of Minas Gerais, based on Index aggregating social, environmental, and epidemiological aspects to downscale climatic scenarios. Since these are densely populated regions that strongly depend on agriculture, the climatic anomalies have the potential to impact the livelihoods and economy of those areas. However, also the northern part of the state even showing less dramatic scenarios of temperature increase and reduction in precipitation shall have an intensification of poverty due to the semiarid climate and high exposure and sensitivity to the drought phenomenon.

The results obtained provide a basis for the decision makers in regard to the need for the targeting of public policies to specific sectors in order to improve the adaptive capacity of the municipalities.

## Figures and Tables

**Table 1:** Components of the IVH Index.

Index of Human Vulnerability (IHV) $IHV=\frac{IGV+CI}{2}$			
*Index of General Vulnerability (IGV)* $IGV=\frac{IEx+ISe+IAC}{3}$			
*Index of Exposure (IEx)* $IEx=\frac{IVC+IND}{2}$	*Indicator of Vegetation Cover (IVC)* $IVC=\frac{Abs\;Area+Re\;latArea}{2}$	(i) Natural vegetation cover (absolute area) (ii) Natural Vegetation cover (percentage)	Source of data: State Institute of Forests of Minas Gerais (IEF)
	*Indicator of Natural Disasters (IND)* $IND=\frac{NaturalDesasters+DesastersDeaths}{2}$	(i) Natural disasters (percentage) (ii) Deaths due to natural disasters (percentage)	Source of data: National Civil Defense (SEDEC)
*Index of Sensitivity (ISe)* $ISe=\frac{IH+IPo+SDI}{3}$	*Index of Health (IH)* $IH=\frac{Dengue+Esquist+Leptos+LTA+Calazar}{5}$	(i) Endemic diseases (incidence) (ii) Endemic diseases (proportion)	Source of data: Ministry of Health (DATASUS)
	*Index of Poverty (IPo)* $IPo=\frac{Mort40y+PopIllit25y+Sanitation+Mort5y+Income}{5}$	(i) Population likely to die before age 40 (proportion) (ii) Population over 25 illiterate (rate) (iii) Households with inadequate sanitation (proportion) (iv) Infant mortality until 5 years old (rate) (v) Population with income below the poverty line (rate)	Source of data: Brazilian Institute of Geography and Statistics (IBGE)
	*Sociodemographic Index (SDI)* $SDI=\frac{FemaleHeadsHouse+HeadsHouseY+Children+Ederly}{4}$	(i) Female heads of household with less than 4 years of education (rate) (ii) Young breadwinners (10–29 years) (rate) (iii) Children up to 5 years (rate) (iv) Elderly population (60 or more) (rate)	Source of data: Brazilian Institute of Geography and Statistics
*Index of Adaptive Capacity (IAC)* $IAC=\frac{FIMD+IPHCC}{2}$	*FIRJAN Index of Municipal Development (FIMD)*	(i) FIMD results	Source of data: Federation of Industries of the State of Rio de Janeiro
	*Indicator of Primary Health Care Coverage (IPHCC)*	(i) Population covered by the primary health care (percentage)	Source of data: Ministry of Health

*Climate Index (CI)* ${CI}_{Scenario}=\frac{AnomTemp+AnomPrec}{2}$		(i) Anomalies of Precipitation and Temperature, Climate Scenario *RCP 4.5*	Source of data: Brazilian Institute for Space Research (INPE)
		(ii) Anomalies of Precipitation and Temperature, Climate Scenario *RCP 8.5*	Source of data: Brazilian Institute for Space Research

Source: elaborated by the author.
